# Supplementary figures and images for: A method to measure molecular hybridization
Source: PLoS One. 2024 Aug 16;19(8):e0308084. doi: 10.1371/journal.pone.0308084 (PMC11329138; doi:10.1371/journal.pone.0308084)

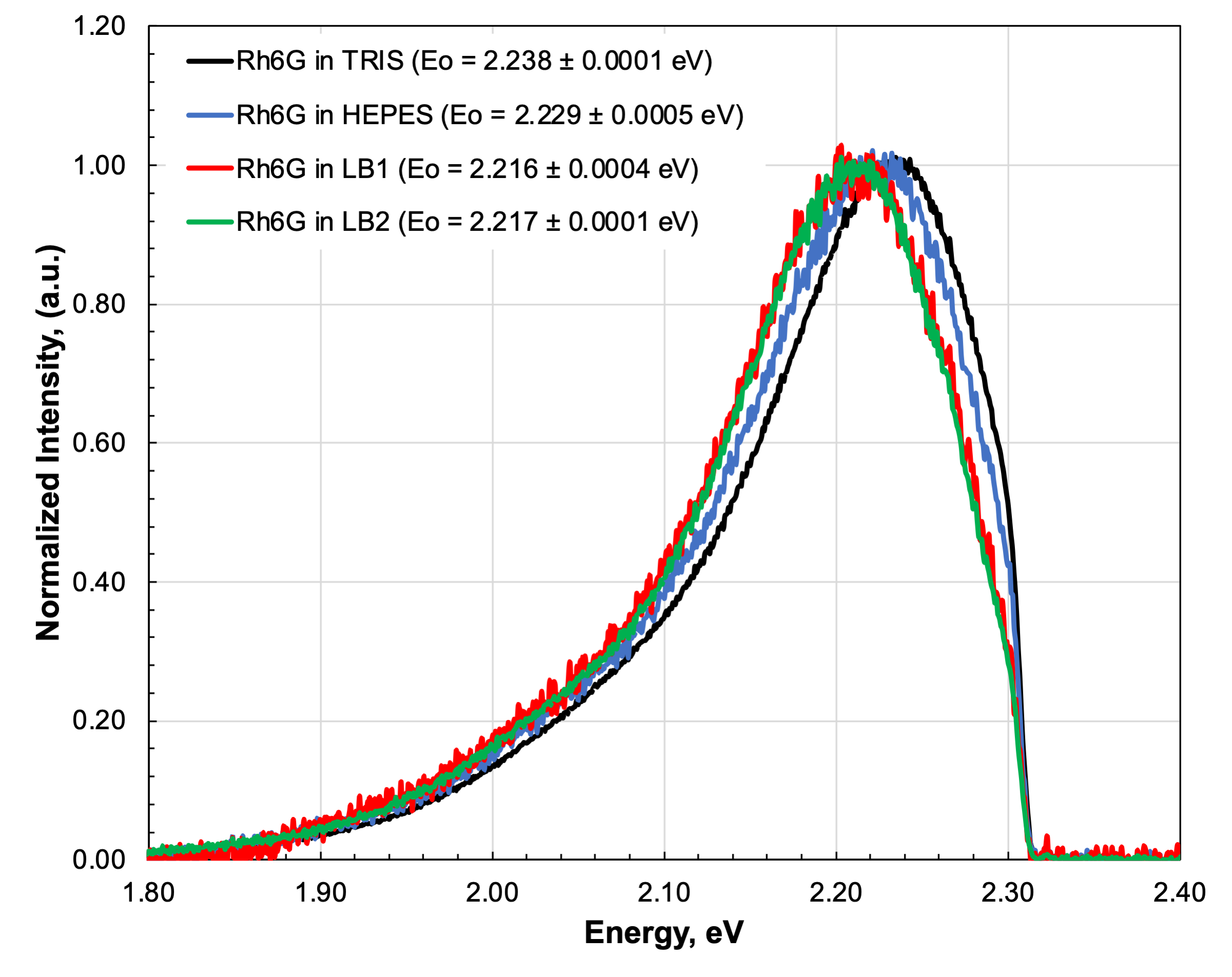

Supplement: S1 Fig — The buffers are Tris-HCl (black), HEPES (blue), LB1 (containing guanidinium thiocyanate) (red) and LB2 (containing Triton X-100) (green). (TIF) [file pone.0308084.s001.tif]

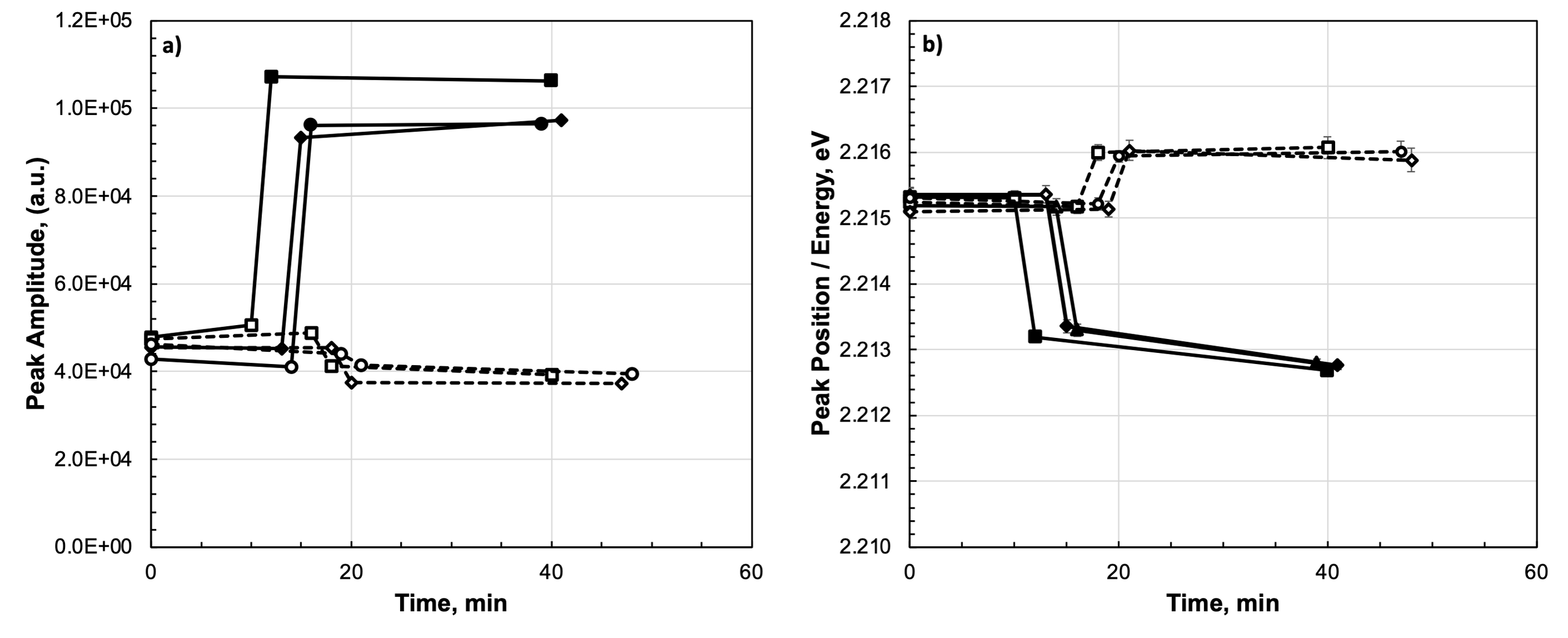

Supplement: S2 Fig — Fluorescence peak amplitude (a) and peak position (b) of the molecular beacon 5’-CGCGATC ATTACTTATAGGGATGGCTATC GATCGCG-3’ in LB1 without (open points and dashed lines) and with complementary strand (solid points and solid lines). Each data point is accompanied by its corresponding standard deviation bars. (TIF) [file pone.0308084.s002.tif]

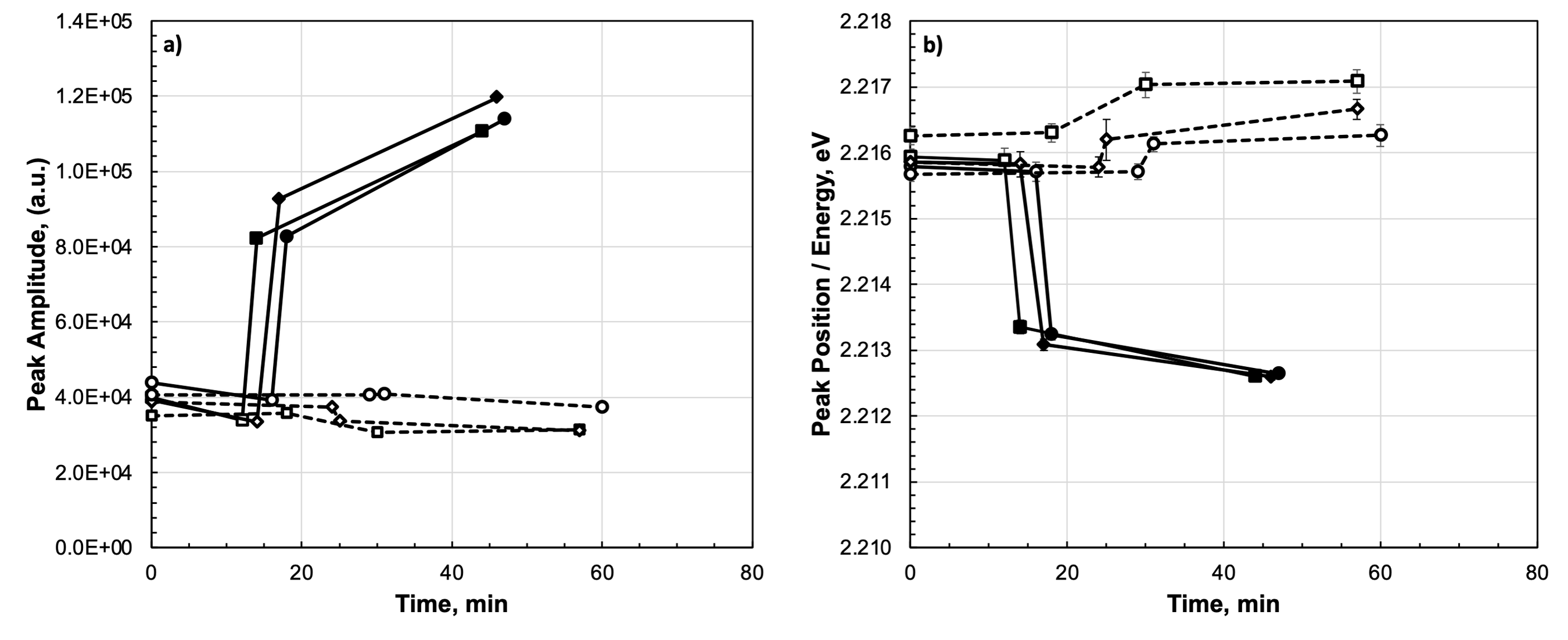

Supplement: S3 Fig — Fluorescence peak amplitude (a) and peak position (b) of the molecular beacon 5’-CGCGATC AAATGCCAGTGTTATCC GATCGCG-3’ in LB1 without (open points and dashed lines) and with complementary strand (solid points and solid lines). Each data point is accompanied by its corresponding standard deviation bars. (TIF) [file pone.0308084.s003.tif]

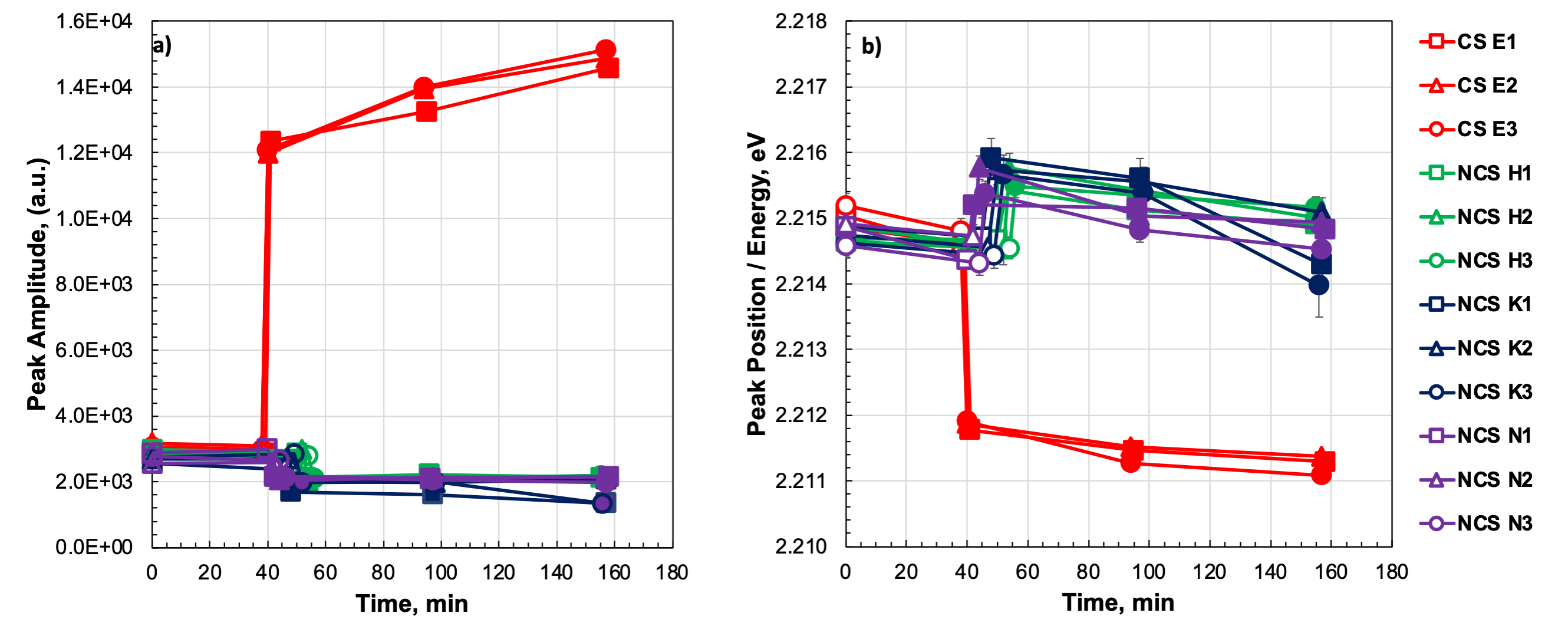

Supplement: S4 Fig — Fluorescence peak amplitude (a) and peak position (b) of the molecular beacon 5′-CGCGAT TAGAGTTCCTGATCTTCTGGTCT ATCGCG-3’ in LB1 without (open points) and with (solid points) target strand. Red color corresponds to the samples with complementary strand (CS). Green, dark blue and violet color to the samples with non-complementary strands (NCS). Each data point is accompanied by its corresponding standard deviation bars. (TIF) [file pone.0308084.s004.tif]
