# Supplementary material for: A method to measure molecular hybridization
Source: PLoS One. 2024 Aug 16;19(8):e0308084. doi: 10.1371/journal.pone.0308084 (PMC11329138; doi:10.1371/journal.pone.0308084)
Supplement: S1 File — (DOCX) [file pone.0308084.s005.docx]

1. Measured fluorescence spectrum $I\left( \lambda\right)$ to be normalized so that the spectrum integration time is equal to $1 sec$.
2. From the spectrum $I\left( \lambda\right)$ background offset must be subtracted
3. Calculate total number of counts $N$ in the spectrum, Equation (1)

$N=\int_{\lambda1}^{\lambda2} I\left( \lambda\right)d\lambda=\int_{E1}^{E2} I\left( E \right)dE\sim\sum_{i} I_{i}$. *(1)*

1. From spectrometer specification find detector sensitivity $\eta$ - number of photons per 1 count and calculate number of photons $\Phi_{o}$ measured by the spectrometer detector:

$\Phi_{o}=\eta N$. *(2)*

1. Considering optical losses of each component $\alpha_{i}$, calculate number of photons collected in the liquid

$\Phi=\prod_{i} \frac{1}{\alpha_{i}}\Phi_{o}$. *(3)*

1. Each dye molecule can generate the following number of photons:

$p=\xi\frac{1}{\tau}$, *(4)*

where $\xi=95\%$ corresponds to the dye quantum efficiency and $\tau\sim4 ns$ - luminescent lifetime

1. Calculate number of molecules $n$ in the working volume

$n=\frac{\Phi}{\gamma p}$, *(3)*

where $\gamma$ corresponds to coupling efficiency, ratio of photons collected by the setup to the total amount of photons generated by a dye molecule. The value can be obtained from ray tracing simulation.
